# C7 genotype of the donor may predict early bacterial infection after liver transplantation

**DOI:** 10.1038/srep24121

**Published:** 2016-04-11

**Authors:** Lin Zhong, Hao Li, Zhiqiang Li, Baojie Shi, PuSen Wang, ChunGuang Wang, Junwei Fan, Hongcheng Sun, Peiwen Wang, Xuebin Qin, Zhihai Peng

**Affiliations:** 1 Department of General Surgery, Shanghai Jiao Tong University Affiliated First People’s Hospital, 85 Wu Jing Road, 200080, China; 2 Bio-X Institutes, Key Laboratory for the Genetics of Developmental and Neuropsychiatric Disorders, Shanghai Jiao Tong University, Shanghai, China; 3 Department of Gastroenterology, Shanghai Jiao Tong University Affiliated First People’s Hospital, 85 Wu Jing Road, 200080, China; 4 Department of Neuroscience, Temple University School of Medicine, Philadelphia, PA 19140, USA

## Abstract

Post-transplantation infection causes high mortality and remains a significant challenge. High clinical risk factors for bacterial infection in recipients are often found in critically ill patients. However, for some recipients, bacterial infections are inevitable. It is conceivable that this susceptibility may be related to the genetics of the donor and recipient. Using expression quantitative trait loci (eQTL) analysis, we found that the C7 rs6876739 CC genotypes and mannan-binding lectin (MBL2) gene polymorphisms of liver donors were significantly associated with bacterial infection in recipients. In an extended validation group of 113 patients, donor C7 rs6876739 genetic variation was an independent risk factor for bacterial infection. The donor C7 rs6876739 CC genotype was associated with lower levels of recipient C7 protein, soluble membrane attack complex (MAC), and IL-1β expression compared with the donor C7 rs6876739 TT genotype. *In vitro*, the MAC significantly triggered NLRP3 inflammasome activation and IL-1β release, suggesting that the mechanism by which C7 defends against bacteria may involve MAC formation, leading to NLRP3 inflammasome activation and IL-1β release. Our findings may be helpful in identifying transplantation recipients at risk of bacterial infection prior to surgery and may contribute to novel infection prevention strategies and the improvement of postoperative outcomes.

Owing to improved surgical techniques and new immunosuppressive drugs, recipient survival after liver transplantation (LT) has increased steadily, with a current 5-year survival rate of between 70% and 80%^1^. However, post-transplantation infection causes high mortality and remains a significant challenge; 50–90% of deaths within six months of LT are linked to infection^2^. Bacterial infections are the most frequently (70%) occurring infectious complications post-transplantation, followed by viral (20%) and fungal infections (8%), and are the leading cause of morbidity and mortality in liver transplant recipients^3^,^4^,^5^. Bacterial infections can occur at any time after LT, but most bacterial infections occur less than 6 months after LT^6^. Therefore, identification of risk factors to allow prediction of early bacterial infection is a pivotal measure for improving the survival of LT recipients by facilitating early, specific treatment and clinical combinational interventions.

High-risk factors for bacterial infection in recipients include critical illness, prolonged operation time, overall level of immunosuppression, postoperative care, and technical complexity of the LT surgery, as well as the occurrence of invasive diagnostic procedures within 6 months of transplantation^7^,^8^. In accordance with this, our previous studies also demonstrated that these were high-risk factors for bacterial infection^9^,^10^. Thus, by shortening the operation time, improving postoperative care, appropriately reducing the dose of immunosuppressive drugs, and limiting invasive diagnostic procedures, the incidence of bacterial infection in LT recipients can be significantly reduced. However, for some recipients, bacterial infections are inevitable^11^, which indicates that these patients are inherently susceptible to infection. It is conceivable that this susceptibility may be related to the genetics of the donor or the recipient. Many studies have focused on the role of genetic factors in the recipient, such as IL28β^12^, and TLR4^13^, in relation to infection; however, the impact of donor genetics on infection after LT has been underestimated. After LT, the donor liver replaces the recipient liver to perform a variety of crucial physiological functions, including the detoxification of various metabolites, the production of biochemicals necessary for digestion, and protein synthesis. Specifically, the liver is responsible for biosynthesis of between 80% and 90% of plasma complement components^14^. The complement system plays an important role in mediating both acquired and innate responses against microbial infections^14^. The complement system can be activated by the classical, lectin and alternative pathways. Each of these leads to activation of the terminal pathway and formation of the membrane attack complex (MAC or C5b-9) to lyse foreign entities^14^.

In addition, because the genetic backgrounds of the donor and recipient are different, the functionality of the donor liver in the recipient and interaction of the donor liver with the recipient immune system will play essential roles in the determination of recipient immunity against bacterial infection. In support of this, an early study using specific target gene sequences has demonstrated that donor polymorphisms in the gene encoding mannose-binding lectin (MBL2), a critical component for lectin pathway activation, influence the risk of potentially life-threatening infections after LT^15^,^16^. To fully understand the susceptibility to infection of liver transplant recipients, we investigated the genetic contributions of the donor liver to the incidence of infection after LT in a genome-wide genetic variation analysis of donor liver gene expression; this approach is known as expression quantitative trait loci (eQTL) analysis^17^.

Genome-wide association studies (GWAS) have found that the majority of allelic variants linked with disease states are located in non-coding regions of the genome, suggesting that complex pathologies are primarily influenced by genetic control of gene expression. Such non-coding genomic regions with the capacity to affect specific mRNA expression levels are termed eQTLs^17^, and their characterization may thus facilitate an improved understanding of disease susceptibility and identification of important molecular drivers of pathology. Furthermore, acquisition of the eQTL dataset of the donor liver may extend our understanding of the mechanisms by which donor genetics can influence the etiology of recipient infection. Therefore, using this approach, we analyzed 77 donor livers; 32 of the recipients had an infection within six months of transplantation and the other 45 did not. The most significant differences between these two groups were found in the complement genes. Complement is an important component of the innate immune system and mediates the initial response to infections by pathogenic microorganisms^14^. Subsequently, we confirmed these results in an extended validation group of 113 patients and found that polymorphisms in two complement component genes, C7 (for MAC formation) and MBL2, were significantly associated with bacterial infection. Specifically, we found that the donor C7 rs6876739 CC genotype was associated with lower levels of recipient C7 protein expression, soluble MAC, and IL-1β compared with the donor C7 rs6876739 TT genotype. *In vitro*, the MAC significantly triggered NLRP3 inflammasome activation and IL-1β release, suggesting that C7 might defend against bacteria by inducing MAC formation, leading to NLRP3 inflammasome activation and IL-1β release. Our findings may be helpful in identifying transplantation recipients at risk of bacterial infection prior to surgery and may contribute to novel infection prevention strategies and improvement of postoperative outcomes.

## Results

### Identification of the complement gene SNPs associated with bacterial infection following LT through eQTL analysis of cohort 1

**Figure 1:**
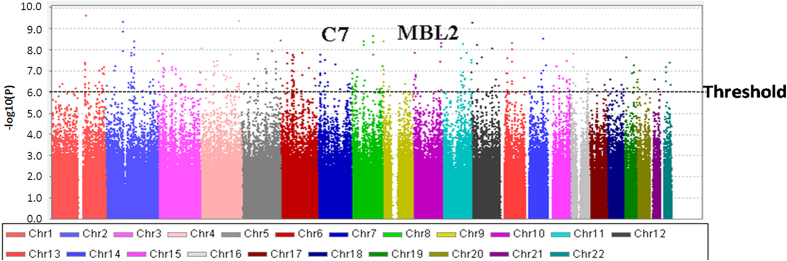
Manhattan plot of genome-wide distribution. Each dot represents an SNP associated with bacterial infection. The SNPs of the C7 and MBL2 genes were significantly associated with bacterial infection (*P* < 10^−6^). SNP, Single nucleotide polymorphism.

We first conducted a genome-wide eQTL analysis of cohort 1 donor livers (n = 77). The characteristics of the patients are shown in Supplementary Table 1. Cohort 1 comprised 77 patients (32 cases with bacterial infection within six months of transplantation and 45 controls without bacterial infection). The main type of bacterial infection was gram-negative bacillus (65.6%). The median age of the patients was 48 years (range 42–55 years), and most patients were male (75.3%). The main reason for LT was hepatocellular carcinoma and cirrhosis related to hepatitis B virus infection (76.6%). After the quality control (QC) process, 367,925 SNPs remained for analysis. In comparing the groups with and without infection, the SNPs most significantly associated with infection were located in complement genes, including C3, C5, C6, C7, C9 and MBL2 (Fig. 1, *P* < 10^−6^). Because complement is an important component of the innate immune system, it provides the initial response to infections by pathogenic microorganisms^14^. Therefore, we chose cohort 2 to confirm and evaluate complement gene SNPs.

### Validation of the association of complement gene SNPs with bacterial infection

The results of our analysis of cohort 1 were validated by assessing the distribution of complement gene SNPs and their association with bacterial infections in cohort 2. Cohort 2 comprised 113 patients: 44 cases with bacterial infection and 69 controls without bacterial infection. The characteristics of the patients are shown in Supplementary Table 2. The median age of these patients was 48 years (range: 42–55 years). Bacterial infections had developed in 44 patients within 6 months of LT. The most common type of infection was gram-negative bacillus, which occurred in 25 (56.8%) patients (Supplementary Table 3). Complement SNPs were detected using Applied Biosystems SNaPshot and TaqMan technology, with *P* < 0.05 considered to indicate statistical significance. Through further analysis, we identified a significant difference in the C7 and MBL2 genotypes between the patient groups with and without infection (Table 1 and Supplementary Table 4). The distribution of the MBL2 genotypes of the donor is shown in Supplementary Table 4. The distribution of rs11003125 was found to be significantly different between the infected and non-infected groups (*P* = 0.024). These results are comparable with findings published previously^15^,^16^. Subsequently, we focused on analyzing the association between C7 gene polymorphisms and bacterial infection after LT. The distribution of the donor C7 gene genotypes is shown in Table 1. rs6876739 was found to be significantly differently distributed between the infected and non-infected groups. The incidence of bacterial infection was significantly higher in patients with the donor rs6876739 CC genotype than in those with the donor rs6876739 TT genotype (34.1% vs. 20.5%, *P* = 0.021). The distribution of the C:T allele frequencies of rs6876739 was 56.8%:43.2% in the infected group compared with 40.6%:59.4% in the uninfected group (*P* = 0.017; Supplementary Table 5). In contrast, we did not find any significant differences when we analyzed the C7 SNP polymorphisms of the recipient and assessed their association with bacterial infection (Supplementary Table 6). In addition, there were no significant differences between the groups in terms of the frequency of distribution of C3, C5, C6, or C9 gene donor polymorphisms (Supplementary Table 7). These results indicate that donor MBL2 and C7 SNPs are associated with bacterial infection in recipients after LT.

### Donor C7 rs6876739 (C allele) improves the predicative value for the risk of bacterial infection in the recipient after LT

We further investigated whether the identified C7 rs6876739 and MBL2 rs11003125 SNPs were independent risk factors for post-surgical bacterial infection. Clinical risk factors for bacterial infection are shown in Supplementary Table 8. The following were identified as risk factors for bacterial infection: postoperative length of stay in the ICU (*P* < 0.001), endotracheal intubation for ≥72 h (*P* = 0.031), post-transplant surgery (*P* = 0.045), and biliary complications (*P* = 0.018). Donor C7 rs6876739 polymorphisms, MBL2 rs11003125 polymorphisms, and clinical parameters (including length of ICU stay after LT, prolonged endotracheal intubation, and biliary complications) were entered into the logistic regression analysis. In multivariate analysis, biliary complications, length of ICU stay after LT, donor MBL2 rs11003125 (G allele, OR = 3.941, *P* = 0.016) and donor C7 rs6876739 (C allele, OR = 4.608, *P* = 0.001) were identified as independent risk factors of bacterial infection after LT (Table 2).

**Figure 2:**
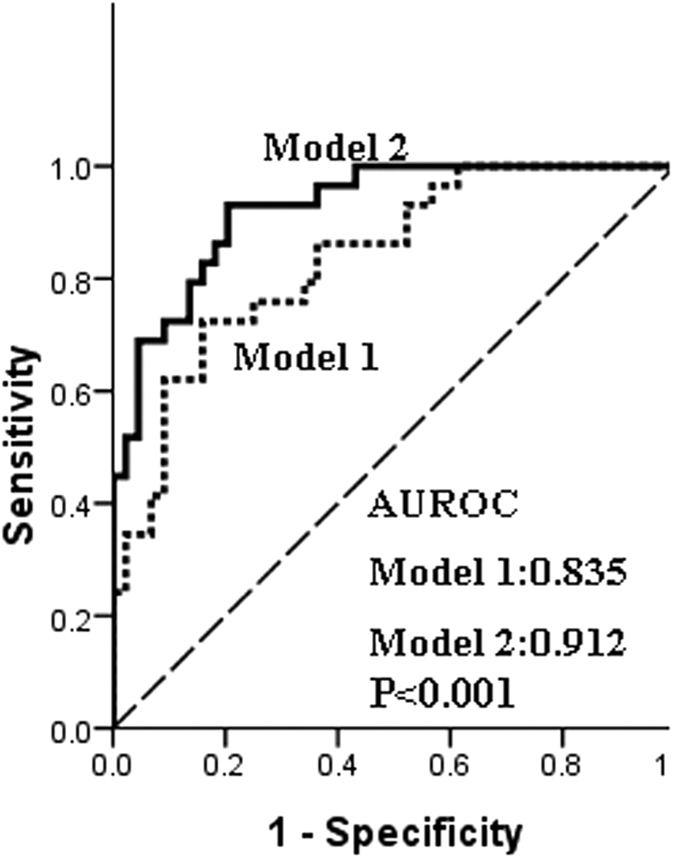
ROC curves of models for the prediction of early bacterial infection. Model 1 included the MBL2 rs11003125 genotype and clinical factors. Model 2 included the C7 rs6876739 genotype based on Model 1. A significant difference was found between Models 1 and 2 (0.835 vs. 0.912, *P* < 0.001). ROC, receiver operating characteristic curve.

Receiver operating characteristic (ROC) curves and areas under the ROC curve (AUC) were used to further evaluate the predictive accuracy of the combination of C7 and MBL2 genotypes and clinical parameters. Figure 2 shows ROC curves of models for the prediction of early bacterial infection. Model 1 included the MBL2 rs11003125 genotype and clinical factors. Model 2 included the C7 rs6876739 genotype and was based on Model 1. In model 1, the AUC was 0.835, which included the donor MBL2 rs11003125 genotype and clinical factors. However, model 2, the combination of the donor C7 rs6876739 genotype, the donor MBL2 rs11003125 genotype, and the clinical factors, had a significantly better predictive value (0.912) than model 1 (*P* < 0.001). This model, based on the donor C7 and MBL genotypes and recipient clinical risks, had relatively good predictive ability and a good fit. The C7 rs6876739 genotype significantly increased the precision of bacterial infection prediction. These results indicate that the C7 rs6876739 (C allele) polymorphism improves the value of the model for predicting the risk of bacterial infection in a recipient after LT.

### Donor C7 rs6876739 CC genotype is associated with lower levels of C7 expression, serum C7 and IL-1β than the donor C7 TT genotype

**Figure 3:**
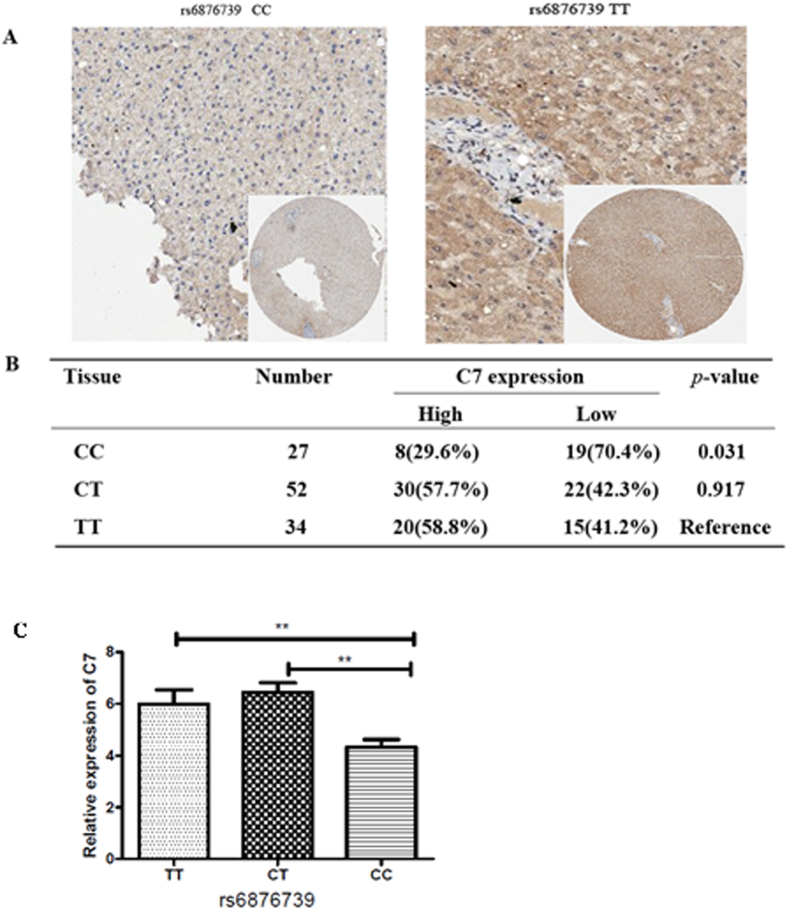
Donor rs6876739 CC liver tissue showed lower levels of C7 mRNA and protein expression than did donor rs6876739 TT liver tissue. (**A**,**B**) Immunohistochemical (IHC) staining of C7 in donor liver tissue with different rs6876739 genotypes. (**A**) Representative IHC images showing C7 staining in donor tissues with the rs6876739 CC and TT genotypes; (**B**) IHC scores of C7 staining in donor liver tissues with the three rs6876739 genotypes. (**C**) The relative expression of C7 in donor liver tissue with different rs6876739 genotypes by RT-PCR.

The value of donor MBL2 gene polymorphisms in predicting bacterial infection after LT has been documented previously^15^,^16^; therefore, we further investigated the molecular and cellular mechanism by which the donor C7 rs6876739 genotype contributes to susceptibility to bacterial infection after LT. We demonstrated that donor livers with the rs6876739 CC genotype expressed lower levels of C7 protein and mRNA than did donor livers with the rs6876739 TT genotype (Fig. 3A–C).

**Figure 4:**
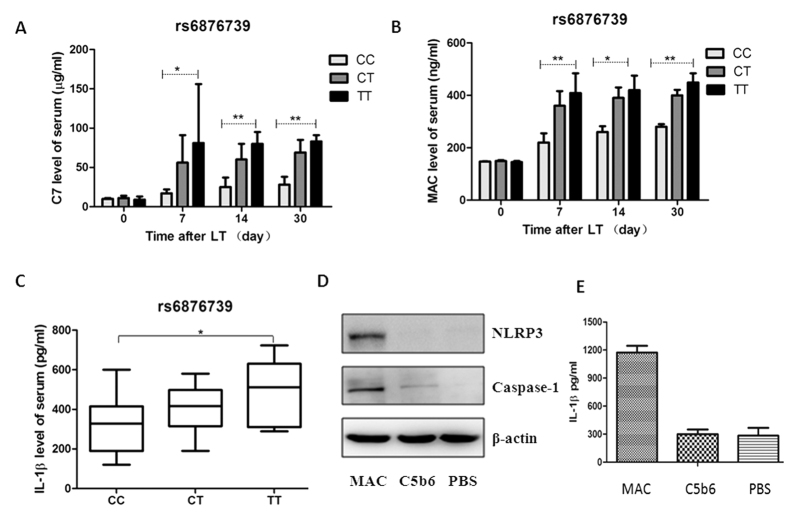
The rs6876739 genotype was associated with C7, soluble MAC and IL-1β release in recipients (**A**–**C**), and MAC activates NLRP3 inflammasomes and IL-1β release *In vitro* (**D**,**E**). (**A**,**B**) The C7 and soluble MAC of recipient were markedly higher in recipients of the donor rs6876739 TT genotype than in those of the donor rs6876739 CC genotype on days 7, 14, 30 after LT. (**C**) The IL-1β levels were markedly higher in recipients of the donor rs6876739 TT genotype than in those of the donor rs6876739 CC genotype (horizontal lines represent the median; the bottom and top of the boxes represent the 25th and 75th percentiles, respectively); (**D**) Western blot analysis showing that MAC stimulates NLRP3 and caspase 1 expression; (**E**) ELISA showing that MAC activates IL-1β release.

To investigate the impact of three different donor liver genotypes (rs6876739 CC, CT and TT) on the serum level of C7 as well as MAC formation in the recipients, we measured serum levels of C7 and soluble MAC (indicative of MAC formation) in recipients (Fig. 4A,B). We found that recipients of livers with the donor rs6876739 CC genotype had significantly lower levels of serum C7 and soluble MAC than did recipients of livers with the rs6876739 TT genotype on days 7, 14, and 30 after transplantation (P < 0 .01) (Fig. 4A,B).

Previous studies have indicated that MAC formation increases the release of IL-1β by activating caspase 1, leading to inflammasome activation^18^; therefore, we measured the levels of soluble MAC and serum IL-1β in the recipients. Recipients of livers with the rs6876739 CC genotype had significantly lower serum IL-1β levels than did recipients of livers with the rs6876739 TT genotype (*P* = 0.022, Fig. 4C). These results, together with previously published findings^18^, indicate that the higher incidence of bacterial infections among recipients of rs6876739 CC genotype livers than among recipients of rs6876739 TT genotype livers may be related to lower MAC formation and IL-1β release.

We further investigated the molecular mechanism by which MAC mediates the release of IL-1β. MAC induced significantly higher levels of caspase 1 and NLRP3 in THP1 cells *In vitro* than did C5b6 alone or PBS (Fig. 4D). MAC also induced significantly higher levels of IL-1β release than did C5b6 alone (1,174 pg/ml vs. 283 pg/ml, *P* < 0.001, Fig. 4E). These results explain our observation that recipients of livers with the rs6876739 CC genotype had lower serum IL-1β levels. The results might indicate the molecular mechanism by which the donor C7 rs6876739 CC genotype increases susceptibility to bacterial infection after LT, although further investigation is required.

## Discussion

Through whole-genome eQTL analysis of SNPs in livers transplanted into recipients who contracted early post-operative bacterial infection and those who did not, we demonstrated that SNPs in the donor complement genes C7 and MBL2 represent risk factors for bacterial infection after LT in a Han Chinese population. We also explored other potential defense mechanisms against pathogen infections in this population.

Complement is an important component of the innate immune system and provides the initial response to infections by pathogenic microorganisms. In our study, we identified a significant association between the donor MBL2 rs11003125 genotype and bacterial infection after LT (*P* = 0.024), with recipients of livers with the MBL2 rs11003125 GG genotype being more prone to infection (OR = 3.941). MBL2, which is a component of the innate immune system, is a serum C-type lectin that generates a rapid response against bacterial infection by binding to carbohydrates on the surface of bacteria^15^,^16^. MBL2 gene polymorphisms were found to be associated with low levels of serum MBL, indicating that the donor MBL2 rs11003125 genotype is predictive of an increased risk of bacterial infection in recipients after LT. These results are consistent with those of other recent studies of LT patients^15^,^16^.

In the present study, we demonstrate that the C7 rs6876739 genotype of the donor rather than the recipient is of greater value in predicting the risk of bacterial infections among recipients after LT. This may be due to the important role of the transplanted liver in C7 synthesis. After LT, the donor liver replaces the recipient liver in performing a variety of crucial physiological functions. Specifically, the liver is a major organ responsible for synthesizing plasma complement components^14^.

In our study, the tendency to develop bacterial infection was greater in patients who received donor livers with the rs6876739 CC genotype than in those who received donor livers with the rs6876739 TT genotype (55.6% vs. 26.5%, *P* = 0.021); the C allele frequency of the rs6876739 polymorphism was more common in livers donated to infected patients than non-infected patients (C:T, 56.8%:43.2% vs. 40.6%:59.4%, *P* = 0.017). However, we did not find that the C7 gene polymorphism of a recipient could predict early bacterial infection after liver transplantation in the clinical samples analyzed here. In the multivariate analysis, donor rs6876739 genotype was identified as an independent risk factor of bacterial infection after LT (OR = 4.608, *P* = 0.001). Recipients of livers with the rs6876739 CC genotype had a more than four-fold increased infection rate compared with recipients of livers with the non-CC genotype. These results suggest that transplantation of livers from donors with the C7 rs6876739 CC genotype could be critical in developing strategies to prevent and treat bacterial infection after LT. Our results also show that the AUROC curve for a model containing the donor rs6876739 genotype provides significantly better predictive ability than a model without the donor rs6876739 genotype (0.912 vs. 0.835, *P* < 0.001), suggesting that the C7 rs6876739 (C allele) improves the value of this model for predicting the risk of bacterial infection in recipients after LT.

It is unclear why recipients of livers with the C7 rs6876739 CC genotype were more prone to infection than were recipients of livers with the rs6876739 TT genotype. We found that C7 protein (immunoreactivity staining score) and mRNA levels were lower in tissue from livers with the rs6876739 CC genotype than from those with the TT genotype (*P* = 0.031 and *P* = 0.017, respectively) and that s6876739 T→C leads to decreased C7 protein expression. C7 is one of the five terminal complement proteins (C5, C6, C7, C8, and C9) that interact sequentially to form a large protein-protein complex, called the MAC, following activation of the complement cascade via the classical, alternative, or lectin pathways. The MAC lyses foreign pathogens directly^14^; hence, C7 deficiency can affect the formation of the MAC, resulting in increased susceptibility to infection^19^,^20^,^21^. Thus, this mechanism may account for the increased susceptibility of recipients of livers with the rs6876739 CC genotype to infection after LT.

The NLRP3 inflammasome and IL-1β are also critical for host defense against bacterial pathogens^22^. It is currently unknown whether the MAC triggers NLRP3 and IL-1β activation to defend against infection in liver recipients. In our study, we found that recipients of livers with the rs6876739 CC genotype had lower serum IL-1β than did recipients of livers with the rs6876739 CC genotype (*P* = 0.022). *In vitro*, we found that MAC activated NLRP3 and IL-1β release in THP1 cells. This was consistent with results reported by Triantafilou *et al*., which showed that MAC triggered intracellular Ca^2+^ flux, leading to NLRP3 inflammasome activation, caspase 1 activation and IL-1β production to eliminate pathogens^18^. Decreased MAC formation may be associated with reduced IL-1β production through activation of caspase 1, followed by inflammasome formation in the recipients; this might further contribute to the high risk of infection in these recipients.

Two limitations of this study should be acknowledged. First, the predictive value of the donor rs6876739 genotype for the risk of infection after LT requires further evaluation in other larger cohort studies. Second, we used only the THP1 cell line to conduct mechanistic studies to highlight the potential role of MAC-induced inflammatory formation during the pathogenesis of infection after LT. This issue requires further investigation with more physiologically relevant experiments conducted using fresh monocyte samples collected from patients after LT.

In conclusion, our findings identified donor C7 gene polymorphisms as an important independent predictor of early bacterial infection and mortality in LT patients. The donor rs6876739 CC genotype was associated with a four-fold increase in relative risk of post-transplant bacterial infection. The donor rs6876739 genotype was implicated as a novel marker of the risk of developing potentially serious bacterial infection after liver transplantation.

## Materials and Methods

### Patients

This study was conducted in two independent cohorts of liver transplantation patients. Cohort 1 comprised 77 donor liver tissue samples for eQTL analysis. Cohort 2 comprised samples from 113 patients for validation of significant results. All patients underwent orthotopic liver transplantation in our transplant center between July 2007 and January 2011. All patients were aged 18 years or older, were blood- and tissue type-compatible with their donors, and did not undergo combined liver/kidney transplantation. The population in our study was ethnically Han Chinese.

### Ethics statement

Written informed consent was obtained from all donors and recipients. All organ donations or transplantations were approved by the Institutional Review Board, Shanghai Jiaotong University Affiliated First Peoples Hospital (China), and carried out strictly in accordance with the guidelines of the Ethics Committee of the hospital and the Declaration of Helsinki^23^. All LT recipients were evaluated using the United Network for Organ Sharing Model for End-Stage Liver Disease (UNOS MELD) scoring system^24^. None of the donor livers were obtained from executed prisoners.

### Definition of infection

LT recipients were divided into two groups: cases with bacterial infection and controls without bacterial infection. Bacterial infection was defined as described previously^9^,^10^. The identified infections were considered clinically significant bacterial infections according to the Centers for Disease Control and Prevention criteria for diagnosing infection^25^. All infections found could be categorized as sepsis, pneumonia, wound infections, peritonitis, urinary tract infections or cholangitis. Bacterial cultures from urine, blood, wound, and intra-abdominal drainages were prepared when infection was suspected.

The diagnosis of bacterial infection was based on fever (>38 °C), elevation of C-reactive protein, specific clinical symptoms of infection as shown below, and a positive bacterial culture.

Sepsis: fever, low arterial blood pressure, systemic inflammatory response, and a positive bacterial blood culture. Wound infections: detection of pus in the wound and a positive bacterial culture. Pneumonia: fever, cough, dyspnea, reduced arterial oxygen, typical pulmonary infiltrate on chest x-ray, and a positive culture from sputum or bronchoalveolar lavage. Urinary tract infection: dysuria, leukocyturia, and a positive urine culture with >10^5^ colony-forming units/ml. Cholangitis: fever, elevation of cholestatic enzymes, and dilated bile ducts detected by ultrasound.

### Reagents

Rabbit polyclonal anti-caspase 1 antibody, rabbit polyclonal anti-NLRP3 antibody and anti-C7 antibody were purchased from Abcam (Cambridge, MA, USA). Human ELISA kits from eBioscience (San Diego, USA) specific for the measurement of IL-1β, C7 and MAC (plates pre-coated with a capture antibody) were used according to the manufacturer’s recommendations. Purified human complement protein C5b6 (0.2 mg/ml), C7, C8, and C9 (1 mg/ml) were purchased from Complement Technology, Inc. (Tyler, TX). Gelatin Veronal-buffered saline (GVB++), which was also purchased from Complement Technology, was used to dilute C5b6, C7, C8 and C9.

### Cells

THP1 cells (a human monocytic cell line derived from an acute monocytic leukemia patient) were purchased from the Chinese Academy of Sciences and cultured in a flask in RPMI-1640 containing 10% fetal bovine serum (Gibco, Carlsbad, CA, USA).

### DNA extraction, GWAS genotyping, and quality control

Genomic DNA was extracted from the EDTA-anti-coagulated whole blood of recipients using a QIAamp DNA Blood Mini Kit (Qiagen, Valencia, CA, USA) and from recently frozen liver tissue from donors using a Maxwell 16 Tissue DNA Purification Kit (Promega, Madison, WI, USA) in accordance with the manufacturers’ instructions. The genome-wide scan was performed using the Affymetrix Genome-Wide Human Single nucleotide-polymorphism (SNP) Array 6.0 for cohort 1. Quality control (QC) filtering of the GWAS data was performed by excluding arrays with a contrast QC < 0.4 from further data analysis. SNPs showing a significant difference between the two groups were validated in cohort 2, and the genotypes of the SNPs were determined using an iPLEX Gold assay on a MassARRAY platform (Sequenom, USA).

### C7 immunohistochemical staining

A rabbit polyclonal anti-human C7 antibody (1:250 dilution; Abcam, Cambridge, MA, USA) was used to detect C7. Staining intensity was graded as follows: 0, none; 1+, mild; 2+, moderate; and 3+, intense. The staining area was scored using the following scale: 0, no staining of cells; 1+, < 10% of tissue stained positive; 2+, 10–50% stained positive; and 3+, >50% stained positive. The level of expression was indicated by the final staining score (i.e., the sum of the intensity and area scores) as follows: 0–2, negative; 3–4, weak; and 5–6, strong.

### C7 cloning by RT-PCR

Total hepatic RNA was isolated from the liver tissue of donors using TRIzol reagent (Gibco BRL) and was reverse transcribed using a High-capacity cDNA Archive Kit (Applied Biosystems, Foster City, CA, USA) according to the manufacturer’s instructions. The full-length human C7 cDNA was amplified by PCR using Pfu DNA polymerase combined with Taq DNA polymerase and specific primers (5′-CTGCGTTGGAGAAACGACAG-3′ and 5′-CCATCTTTCAAGGCAGGAGG-3′).

### C7, soluble MAC and IL-1β ELISAs

The recipients were divided into three groups according to donor C7 genotype: rs6876739 TT, CT and CC. The C7 and soluble MAC levels in the serum of recipients were tested at the indicated time (preoperative, 7^th^, 14^th^, 30^th^ day) after LT, and IL-1β levels were tested on the 7^th^ postoperative day by ELISA, according to the manufacturer’s recommendations (eBioscience, San Diego, USA).

### *In vitro* assay for MAC-mediated inflammasome activation

Approximately 5 × 10^5^ THP1 cells per well were seeded on 6-well plates and incubated overnight at 37 °C in 5% CO_2_. The culture medium was removed, and 1 ml of RPMI-1640 with 5% FBS was added. The cells were stimulated with MAC as follows: GVB buffer-diluted C5b6 (final concentration 25 μg/ml) was added to the plates, followed by C7, C8 and C9 (final concentration 25 μg/ml). The THP1 cells were stimulated with C5b6 or PBS as a control.

After 12h stimulation, the production of IL-1β was quantified in the culture supernatants by ELISA. Total cell proteins were isolated, and protein lysates (15 μg per lane) were resolved by 10% sodium dodecyl sulfate-polyacrylamide gel electrophoresis (SDS-PAGE) and transferred to nitrocellulose membranes. For Western blotting, the membranes were incubated with primary anti-NLRP3 and anti-caspase 1 antibodies diluted in blocking buffer for 1 to 2 h at room temperature or overnight at 4 °C and were subsequently incubated with the appropriate secondary antibody.

### Statistical analysis

Quantitative variables were expressed as the mean ± SD and compared using Student’s *t*-test or the Mann–Whitney test. Categorical variables were presented as values (percentages) and compared using the Pearson *χ*^2^ test or Fisher exact test. The genotype frequencies were also determined using the *χ*^2^ test for Hardy–Weinberg equilibrium. Risk factors for bacterial infection were evaluated using logistic regression analysis. Variables with statistical significance in univariate analysis were subsequently investigated using stepwise multivariate regression analysis. SHEsis Online Version (http://analysis.bio-x.cn/myAnalysis.php) was used to analyze linkage disequilibrium. The area under the receiver operating characteristic curve (AUROC) was calculated to evaluate the ability of a model to predict bacterial infection. *P* values less than 0.05 were considered to indicate statistical significance for all statistical comparisons. Statistical analyses were performed using SPSS 19.0 software (SPSS, Chicago, IL, USA).

## Additional Information

**How to cite this article**: Zhong, L. *et al*. C7 genotype of the donor may predict early bacterial infection after liver transplantation. *Sci. Rep.* **6**, 24121; doi: 10.1038/srep24121 (2016).

## Supplementary Material

Supplementary Information

## Figures and Tables

**Table 1:** Donor C7 genotype distribution and association with bacterial infection.

**SNP**	**Genotype distribution, n (%)**		**P value**^**b**^	**HWE P value**
	**Infected (44)**	**Non-infected (69)**		
Donor				
rs6876739				0.625
Genotype				
CC	15 (34.1%)	12 (17.4%)	0.021	
CT	20 (45.5%)	32 (46.4%)	0.250	
TT	9 (20.5%)	25 (36.2%)		

Abbreviations: SNP, Single nucleotide polymorphism; HWE, Hardy-Weinberg equilibrium.

**Table 2:** Risk factors associated with infection.

	**Odds ratio (95% CI)**	**P value**
**Model 1 (MBL2 with clinical parameters)**		
Biliary complications (ref = no)	9.990 (1.423–70.135)	0.021
ICU stay after LT (h)	1.004 (1.001–1.007)	0.043
Donor MBL2 rs11003125 genotype (0 = CC, 1 = CG, 2 = GG)	3.941 (2.076-7.725)	0.016
**Model 2 (C7 with Model 1)**		
Donor C7 rs6876739 genotype (0 = TT, 1 = CT, 2 = CC)	4.608 (1.938–10.989)	0.001
