# Supplementary material for: C7 genotype of the donor may predict early bacterial infection after liver transplantation
Source: Sci Rep. 2016 Apr 11;6:24121. doi: 10.1038/srep24121 (PMC4827091; doi:10.1038/srep24121)
Supplement: Supplementary Information [file srep24121-s1.doc]

**C7** **genotype of the donor** **may predict** **early bacterial infection after liver transplantation**

Lin Zhong^1*^, Hao Li^1*^, Zhiqiang Li^2^, Baojie Shi^1^, PuShen Wang^1^, ChunGuang Wang^1^, Junwei Fan^1^, Hongcheng Sun^1^, Peiwen Wang^3^, Xuebin Qin^4^, Zhihai Peng^1^

^1^Department of General Surgery, Shanghai Jiao Tong University Affiliated First People's Hospital, 85 Wu Jing Road, 200080, China

^2^Key Laboratory for the Genetics of Developmental and Neuropsychiatric Disorders, Bio-X Institutes, Ministry of Education, Shanghai Jiao Tong University; Shanghai genome Pilot Institutes for Genomics and Human Health, 50 West Guangyuan Road, 200030, China

^3^Department of Gastroenterology, Shanghai Jiao Tong University Affiliated First People's Hospital, 85 Wu Jing Road, 200080, China

^4^Department of Neuroscience, Temple University School of Medicine, Philadelphia, PA 19140, USA.

^*^These authors contributed equally to this work.

*Correspondence to: Prof. Zhi-hai Peng, Department of General Surgery, Shanghai Jiao Tong University Affiliated First People's Hospital, 85 Wu Jing Road, 200080, Shanghai, China. Tel: +86-021-63240090-3133, Fax: +86-021-63242903, E-mail: [pengzhihai81@163.com](mailto:pengzhihai81@163.com). Prof Xue-bin Qin, Department of Neuroscience, Temple University School of Medicine, Philadelphia, PA 19140, USA. E-mail: [xuebin.qin@temple.edu](mailto:xuebin.qin@temple.edu). Prof. Pei-wen Wang, Department of Gastroenterology, Shanghai Jiao Tong University Affiliated First People's Hospital, 85 Wu Jing Road, 200080, Shanghai, China. E-mail: [tjw1135@126.com](mailto:tjw1135@126.com).

**Supplementary Table 1** Summary of 77 patients’ demographic and clinical characteristics

|  | | N=77 |
| --- | --- | --- |
| Preoperative characteristics | |  |
| Age^1^ (year) | | 48.0 (42, 55.0) |
| Gender^2^ | Male | 58 (75.3%) |
|  | Female | 19 (24.7%) |
| Infection status | No infection | 45 (58.4%) |
|  | Infection | 32 (41.6%) |
|  | Gram-positive bacillus | 11 (34.4%) |
|  | Gram-negative bacillus | 21 (65.6%) |
| The reasons for LT | |  |
| Hepatocellular carcinoma | | 39 (50.6%) |
| Cirrhosis related to hepatitis B virus infection | | 20 (26.0%) |
| Other etiologies | | 18 (23.4%) |

Abbreviations: LT, liver transplantation;

^1^Continuous data are presented as median with inter-quartile range (IQR).

**Supplementary Table 2** Summary of patients’ demographic and clinical characteristics

|  | | N=113 |
| --- | --- | --- |
| Preoperative characteristics | |  |
| Age^1^ (year) | | 48.0 (42.0, 55.0) |
| Gender^2^ | Male | 94 (83.2%) |
|  | Female | 19 (16.8%) |
| Infection status^2^ | No infection | 69 (61.1%) |
|  | Infection | 44 (38.9%) |
| The reasons for LT^2^ | |  |
| Hepatocellular carcinoma | | 60 (53.1%) |
| Cirrhosis related to hepatitis B virus infection | | 32 (28.3%) |
| Hepatitis C virus- and alcohol-related cirrhosis | | 4 (3.5%) |
| Autoimmune cirrhosis | | 3 (2.7%) |
| Nonfunctional graft | | 6 (5.3%) |
| Other etiologies | | 8 (7.1%) |
| Operative characteristics | |  |
| Blood loss^1^ (L) | | 2.6 (1.2, 5.4) |
| Packed red cell transfusion^1^ (U) | | 8.0 (2.0, 14.0) |
| Operative time^1^ (h) | | 6.0 (6.0, 8.0) |
| Duration of anhepatic phase^1^ (min) | | 60.0 (55.0, 60.0) |
| Duration of post-transplant nutrition support^1^ (day) | | 3.2 (2.3, 4.3) |
| Duration of stay in ICU after LT^1^ (day) | | 16.0 (10.5, 23.0) |

Abbreviations: LT, liver transplantation;

^1^Continuous data are presented as median with inter-quartile range (IQR).

^2^Categorical data are presented as count and percentage.

**Supplementary Table 3** The main types and source of bacterial infection that patients acquired post-LT.

|  | Gram-positive bacillus (n=19) | Gram-negative bacillus (n=25) |
| --- | --- | --- |
| Type of infection |  |  |
| Pulmonary infection | 8 (42.1%) | 13 (52.0%) |
| Sepsis | 5 (26.3%) | 4 (16.0%) |
| Cholangitis | 4 (21.1%) | 5 (20.0%) |
| Other | 2 (10.5%) | 3 (12.0%) |

**Supplementary Table 4** Donor MBL2 genotype distribution and association with bacterial infection

| SNP | Genotype distribution, n (%) | | *P* value^b^ |
| --- | --- | --- | --- |
|  | Infected (44) | Noninfected (69) |  |
| Donor |  |  |  |
| rs11003125 |  |  |  |
| Genotype |  |  |  |
| CC | 3 (6.8%) | 11 (15.9%) |  |
| CG | 7 (15.9%) | 30 (43.5%) | 1.000 |
| GG | 34 (77.3%) | 28 (40.6%) | 0.024 |

Abbreviations: SNP, Single nucleotide polymorphism; HWE, Hardy-Weinberg equilibrium.

**Supplementary Table 5** Alleles distributions of donor rs6876739 between the control and infection groups

| SNP | Study groups (n) | Allele n (%) | | X^2^ | *P* value | OR (95%CI) |
| --- | --- | --- | --- | --- | --- | --- |
| rs6876739 |  | C | T |  |  |  |
|  | Infection (44) | 50 (56.8%) | 38 (43.2%) |  |  |  |
|  | Control (69) | 56 (40.6%) | 82 (59.4%) | 5.689 | 0.017 | 1.927 (1.121–3.312) |

OR, Odds Ratio; CI, confidence intervals.

**Supplementary Table 6** Recipient C7 genotype distribution and association with bacterial infection

| SNP | Genotype distribution, n(%) | | *P* value^b^ | HWE  P value |
| --- | --- | --- | --- | --- |
|  | Infection | NO infection |  |  |
| Recipient |  |  |  |  |
| rs6876739 |  |  |  | 0.784 |
| genotype |  |  |  |  |
| CC | 7(15.9%) | 18(26.5%) |  |  |
| CT | 20(45.5%) | 30(44.1%) | 0.307 |  |
| TT | 17(38.6%) | 20(29.4%) | 0.155 |  |
| rs16870514 |  |  |  | 0.199 |
| genotype |  |  |  |  |
| AA | 9(20.5%) | 7(10.4%) |  |  |
| AG | 17(38.6%) | 25(37.3%) | 0.280 |  |
| GG | 18(40.9%) | 35(52.2%) | 0.109 |  |
| rs1501815 |  |  |  | 0.070 |
| genotype |  |  |  |  |
| AA | 25(56.8%) | 40(58.8%) | 0.340 |  |
| AT | 13(29.5%) | 23(33.8%) | 0.312 |  |
| TT | 6(13.6%) | 5(7.4%) |  |  |
| rs2597739 |  |  |  | 0.118 |
| genotype |  |  |  |  |
| CC | 24(54.5%) | 27(39.7%) | 0.191 |  |
| CT | 14(31.8%) | 27(39.7%) | 0.746 |  |
| TT | 6(13.6%) | 14(20.6%) |  |  |
| rs2443040 |  |  |  | 0.232 |
| genotype |  |  |  |  |
| GG | 12(27.3%) | 21(30.4%) |  |  |
| GT | 18(40.9%) | 34(49.3%) | 0.180 |  |
| TT | 14(31.8%) | 14(20.3%) | 0.283 |  |
| rs3792642 |  |  |  | 0.682 |
| genotype |  |  |  |  |
| CC | 26(59.1%) | 36(53.7%) | 1.000 |  |
| CT | 15(34.1%) | 27(40.3%) | 0.697 |  |
| TT | 3(6.8%) | 4(6%) |  |  |
| rs9292795 |  |  |  | 0.283 |
| genotype |  |  |  |  |
| AA | 6(13.6%) | 5(7.4%) |  |  |
| AT | 16(36.4%) | 35(51.5%) | 0.176 |  |
| TT | 22(50%) | 28(41.2%) | 0.525 |  |
| rs1450656 |  |  |  | 0.02 |
| genotype |  |  |  |  |
| AA | 27(61.4%) | 30(44.1%) | 0.483 |  |
| AG | 11(25%) | 28(41.2%) | 0.533 |  |
| GG | 6(13.6%) | 10(14.7%) |  |  |
| rs1551090 |  |  |  | 0.187 |
| genotype |  |  |  |  |
| CC | 6(13.6%) | 11(16.2%) |  |  |
| CG | 15(34.1%) | 33(48.5%) | 0.759 |  |
| GG | 23(52.3%) | 24(35.3%) | 0.333 |  |
| Rs2675981 |  |  |  | 0.816 |
| genotype |  |  |  |  |
| AA | 33(75%) | 41(60.3%) | 1.000 |  |
| AG | 10(22.7%) | 26(38.2%) | 0.501 |  |
| GG | 1(2.3%) | 1(1.5%) |  |  |
| rs1901167 |  |  |  | 0.721 |
| CC | 6(13.6%) | 10(14.7%) |  |  |
| CT | 19(43.2%) | 34(50%) | 0.904 |  |
| TT | 19(43.2%) | 24(35.3%) | 0.644 |  |
| rs2329434 |  |  |  | 0.333 |
| AA | 22(50%) | 28(41.2%) | 0.691 |  |
| AT | 20(45.5%) | 36(52.9%) | 1.000 |  |
| TT | 2(4.5%) | 4(5.9%) |  |  |
| rs1078374 |  |  |  | 0.262 |
| AA | 22(62.9%) | 36(59%) | 1.000 |  |
| AG | 10(28.6%) | 21(34.4%) | 0.672 |  |
| GG | 3(8.6%) | 4(6.6%) |  |  |

**Supplementary Table 7** Donor C3, C5, C6, and C9 genotype distribution and association with bacterial infection

| Gene | SNPs | | Genotype distribution, n(%) | | *P* value^b^ | HWE  P value |
| --- | --- | --- | --- | --- | --- | --- |
|  |  |  | Infection | No infection |  |  |
| C3 | Rs2250656 | AA | 22(51.2%) | 46(67.6%) | 0.191 | 0.916 |
|  |  | AG | 18(41.9%) | 20(29.4%) |  |  |
|  |  | GG | 3(7.0%) | 2(2.9%) |  |  |
|  | Rs17030 | AA | 9(20.5%) | 17(24.6%) | 0.685 | 0.397 |
|  |  | AG | 26(59.1%) | 35(50.7%) |  |  |
|  |  | GG | 9(20.5%) | 17(24.6%) |  |  |
|  | Rs10411506 | AA | 12(27.3%) | 15(21.7%) | 0.769 | 0.245 |
|  |  | AG | 18(40.9%) | 32(46.4%) |  |  |
|  |  | GG | 14(31.8%) | 22(31.9%) |  |  |
|  | Rs344555 | AA | 4(9.1%) | 4(5.8%) | 0.792 | 0.884 |
|  |  | AG | 16(36.4%) | 27(39.1%) |  |  |
|  |  | GG | 24(54.5%) | 38(55.1%) |  |  |
|  | Rs11672613 | CC | 9(20.5%) | 13(18.8%) | 0.730 | 0.342 |
|  |  | CT | 21(47.7%) | 29(42.0%) |  |  |
|  |  | TT | 14(31.8%) | 27(39.1%) |  |  |
|  | Rs2241393 | CC | 4(9.1%) | 7(10.1%) | 0.474 | 0.306 |
|  |  | CG | 19(43.2%) | 37(53.6%) |  |  |
|  |  | GG | 21(47.7%) | 25(36.2%) |  |  |
|  | Rs6417195 | AA | 5(11.4%) | 2(2.9%) | 0.160 | 0.975 |
|  |  | AG | 14(31.8%) | 28(40.6%) |  |  |
|  |  | GG | 25(56.8%) | 39(56.5%) |  |  |
| C5 | Rs4837805 | AA | 30(69.8%) | 47(68.1%) | 0.962 | 0.155 |
|  |  | AG | 11(25.6%) | 18(26.1%) |  |  |
|  |  | GG | 2(4.7%) | 4(5.8%) |  |  |
|  | Rs2300929 | CC | 2(4.7%) | 1(1.4%) | 0.294 | 0.713 |
|  |  | CT | 10(23.3%) | 24(34.8%) |  |  |
|  |  | TT | 31(72.1%) | 44(63.8%) |  |  |
|  | Rs25681 | CC | 6(13.6%) | 7(10.1%) | 0.234 | 0.535 |
|  |  | CT | 17(38.6%) | 38(55.1%) |  |  |
|  |  | TT | 21(47.7%) | 24(34.8%) |  |  |
|  | Rs17611 | AA | 15(42.9%) | 22(36.1%) | 0.662 | 0.621 |
|  |  | AG | 15(42.9%) | 32(52.5%) |  |  |
|  |  | GG | 5(14.3%) | 7(11.5%) |  |  |
| C6 | Rs3805712 | AA | 5(11.4%) | 6(8.7%) | 0.745 | 0.734 |
|  |  | AG | 21(47.7%) | 30(43.5%) |  |  |
|  |  | GG | 18(40.9%) | 33(47.8%) |  |  |
|  | Rs6865420 | AA | 3(8.6%) | 4(6.6%) | 0.865 | 0.491 |
|  |  | AC | 11(31.4%) | 22(36.1%) |  |  |
|  |  | CC | 21(60.0%) | 35(57.4%) |  |  |
|  | Rs3805716 | AA | 21(47.7%) | 37(53.6%) | 0.488 | 0.034 |
|  |  | AT | 18(40.9%) | 21(30.4%) |  |  |
|  |  | TT | 5(11.4%) | 11(15.9%) |  |  |
|  | Rs3805715 | AA | 21(47.7%) | 34(49.3%) | 0.970 | 0.987 |
|  |  | AG | 20(45.5%) | 31(44.9%) |  |  |
|  |  | GG | 3(6.8%) | 4(5.8%) |  |  |
|  | Rs9200 | AA | 22(50.0%) | 34(50.0%) | 0.859 | 0.102 |
|  |  | AG | 17(38.6%) | 24(35.3%) |  |  |
|  |  | GG | 5(11.4%) | 10(14.7%) |  |  |
|  | Rs7443562 | AA | 13(29.5%) | 20(29.0%) | 0.949 | 0.143 |
|  |  | AG | 24(54.5%) | 39(56.5%) |  |  |
|  |  | GG | 7(15.9%) | 10(14.5%) |  |  |
|  | Rs10052999 | CC | 18(51.4%) | 38(63.3%) | 0.514 | 0.142 |
|  |  | CT | 16(45.7%) | 21(35.0%) |  |  |
|  |  | TT | 1(2.9%) | 1(1.7%) |  |  |
| C9 | Rs187451 | AA | 23(53.5%) | 39(56.5%) | 0.434 | 0.614 |
|  |  | AC | 19(44.2%) | 25(36.2%) |  |  |
|  |  | CC | 1(2.3%) | 5(7.2%) |  |  |
|  | Rs534663 | CC | 14(32.6%) | 26(37.7%) | 0.242 | 0.666 |
|  |  | CT | 18(41.9%) | 34(49.3%) |  |  |
|  |  | TT | 11(25.6%) | 9(13.0%) |  |  |
|  | Rs155377 | CC | 19(43.2%) | 28(40.6%) | 0.350 | 0.948 |
|  |  | CT | 22(50.0%) | 30(43.5%) |  |  |
|  |  | TT | 3(6.8%) | 11(15.9%) |  |  |
|  | Rs11953839 | AA | 11(25.0%) | 11(15.9%) | 0.154 | 0.577 |
|  |  | AG | 18(40.9%) | 41(59.4%) |  |  |
|  |  | GG | 15(34.1%) | 17(24.6%) |  |  |
|  | Rs644771 | CC | 17(38.6%) | 21(30.4%) | 0.325 | 0.501 |
|  |  | CT | 23(52.3%) | 35(50.7%) |  |  |
|  |  | TT | 4(9.1%) | 13(18.8%) |  |  |
|  | Rs1971419 | AA | 4(9.1%) | 6(8.8%) | 0.969 | 0.680 |
|  |  | AG | 19(43.2%) | 31(45.6%) |  |  |
|  |  | GG | 21(47.7%) | 31(45.6%) |  |  |
|  | Rs11740241 | CC | 0(0%) | 4(5.8%) | 0.226 | 0.505 |
|  |  | CT | 13(29.5%) | 16(23.2%) |  |  |
|  |  | TT | 31(70.5%) | 49(71.0%) |  |  |
|  | Rs476569 | CC | 13(30.2%) | 11(15.9%) | 0.159 | 0.441 |
|  |  | CT | 19(44.2%) | 41(59.4%) |  |  |
|  |  | TT | 11(25.6%) | 17(24.6%) |  |  |
|  | Rs696766 | AA | 5(11.6%) | 8(11.6%) | 0.841 | 0.922 |
|  |  | AG | 21(48.8%) | 30(43.5%) |  |  |
|  |  | GG | 17(39.5%) | 31(44.9%) |  |  |
|  | Rs13179150 | AA | 8(18.2%) | 5(7.2%) | 0.177 | 0.814 |
|  |  | AC | 19(43.2%) | 30(43.5%) |  |  |
|  |  | CC | 17(38.6%) | 34(49.3%) |  |  |
|  | Rs700227 | CC | 22(51.2%) | 35(50.7%) | 0.692 | 0.135 |
|  |  | CG | 17(39.5%) | 24(34.8%) |  |  |
|  |  | GG | 4(9.3%) | 10(14.5%) |  |  |
|  | Rs261753 | CC | 17(38.6%) | 30(43.5%) | 0.861 | 0.738 |
|  |  | CT | 22(50.0%) | 31(44.9%) |  |  |
|  |  | TT | 5(11.4%) | 8(11.6%) |  |  |
|  | Rs4957473 | AA | 30(68.2%) | 38(55.9%) | 0.401 | 0.298 |
|  |  | AG | 11(25.0%) | 25(36.8%) |  |  |
|  |  | GG | 3(6.8%) | 5(7.4%) |  |  |
|  | Rs121909592 | CC | 42(95.5%) | 66(97.1%) | 0.655 | 0.847 |
|  |  | CT | 2(4.5%) | 2(2.9%) |  |  |
|  |  | TT |  |  |  |  |
|  | Rs696764 | AA | 4(9.1%) | 3(4.4%) | 0.535 | 0.018 |
|  |  | AG | 22(50.0%) | 39(57.4%) |  |  |
|  |  | GG | 18(40.9%) | 26(38.2%) |  |  |
|  | Rs835219 | CC | 6(14.0%) | 10(14.7%) | 0.506 | 0.965 |
|  |  | CT | 23(53.5%) | 29(42.6%) |  |  |
|  |  | TT | 14(32.6%) | 29(42.6%) |  |  |

**Supplementary Table 8** Demographic and clinical characteristics distributions of subjects

|  | Total  (N=113) | Non-infected (N=69) | Infected  (N=44) | *P*-value |
| --- | --- | --- | --- | --- |
| Pre-transplant variables |  |  |  |  |
| Age (years) | 48 (42, 55) | 48 (42, 54) | 49 (41.5, 55) | 0.748 |
| Gender |  |  |  | 0.018* |
| Male | 94 (83.19%) | 62 (89.86%) | 32 (72.73%) |  |
| Female | 19 (16.81%) | 7 (10.14%) | 12 (27.27%) |  |
| MELD score | 10 (8, 14) | 10 (8, 13) | 11 (8, 14) | 0.410 |
| Encephalopathy gradesⅡ-Ⅳ | 5 (4.4%) | 3 (4.4%) | 2 (4.55%) | 0.960 |
| Child-Pugh score | 7 (5, 9) | 7 (5, 8) | 7 (5, 9) | 0.599 |
| Diabetes mellitus | 8 (7.08%) | 6 (8.7%) | 2 (4.55%) | 0.480 |
| Reasons for LT |  |  |  |  |
| Hepatocellular carcinoma | 60 (53.1%) | 40 (57.97%) | 20 (45.45%) | 0.386 |
| Cirrhosis related to hepatitis B viruses | 33 (29.2%) | 21 (30.43%) | 12 (27.27%) |  |
| Hepatitis C virus- and alcohol-related cirrhosis | 3 (2.65%) | 1 (1.45%) | 2 (4.55%) |  |
| Autoimmune cirrhosis | 4 (3.54%) | 2 (2.9%) | 2 (4.55%) |  |
| Non-functioning graft | 6 (5.31%) | 2 (2.9%) | 4 (9.09%) |  |
| Other etiologies | 7 (6.19%) | 3 (4.35%) | 4 (9.09%) |  |
| Operative variables |  |  |  |  |
| Packed red cell transfusion (U) | 8 (2, 14) | 6 (0, 12) | 9 (5, 14) | 0.243 |
| Blood loss during LT (mL) | 2550 (1200, 5250) | 2550 (1300, 5000) | 2750 (1200, 6250) | 0.672 |
| Operation time (hrs) | 6 (6, 8) | 6 (5.5, 7) | 6.5 (6, 8) | 0.096 |
| Anhepatic time (min) | 60 (55, 60) | 60 (50, 60) | 60 (59, 60) | 0.450 |
| Post-transplant variables |  |  |  |  |
| Post-LT transfusion (U) | 0 (0, 4) | 0 (0, 3) | 0 (0, 7.5) | 0.122 |
| ICU stay after LT (hrs) | 384 (264, 552) | 312 (216, 480) | 504 (336, 648) | <0.001* |
| Post-transplant renal dysfunction | 7 (6.19%) | 3 (4.35%) | 4 (9.09%) | 0.428 |
| Prolonged endotracheal intubation (≥72 hrs) | 22 (19.47%) | 9 (13.04%) | 13 (29.55%) | 0.031* |
| Endotracheal reintubation | 5 (4.42%) | 1 (1.45%) | 4 (9.09%) | 0.075 |
| Post-transplant reoperative episodes | 10 (8.85%) | 3 (4.35%) | 7 (15.91%) | 0.045* |
| Acute rejection with high-dose corticosteroids or ATG therapy | 20 (17.7%) | 11 (15.94%) | 9 (20.45%) | 0.540 |
| Biliary complications | 15 (13.27%) | 5 (7.25%) | 10 (22.73%) | 0.018* |
| Post-LT without application of prednisone | 32 (33.33%) | 16 (26.23%) | 16 (45.71%) | 0.051 |

Abbreviations: MELD, model for end stage liver disease; LT, liver transplantation; ATG, anti-thymocyte globulin.

Continuous variables are presented as median and interquartile ranges (IQRs). Categorical variables are presented as counts and percentages.

* P<0.05, significant differences between the infected and non-infected groups.
